# Structure-function analysis of Sedolisins: evolution of tripeptidyl peptidase and endopeptidase subfamilies in fungi

**DOI:** 10.1186/s12859-018-2404-y

**Published:** 2018-12-04

**Authors:** Facundo Orts, Arjen ten Have

**Affiliations:** Instituto de Investigaciones Biológicas (IIB-CONICET-UNMdP), Facultad de Ciencias Exactas y Naturales, Universidad Nacional de Mar del Plata, CC 1245, 7600 Mar del Plata, Argentina

**Keywords:** Functional redundancy and diversification, Structure-function analysis, Protein superfamily, Mutual Infomation, Protease, Subtilisin

## Abstract

**Background:**

Sedolisins are acid proteases that are related to the basic subtilisins. They have been identified in all three superkingdoms but are not ubiquitous, although fungi that secrete acids as part of their lifestyle can have up to six paralogs. Both TriPeptidyl Peptidase (TPP) and endopeptidase activity have been identified and it has been suggested that these correspond to separate subfamilies.

**Results:**

We studied eukaryotic sedolisins by computational analysis. A maximum likelihood tree shows one major clade containing non-fungal sequences only and two major as well as two minor clades containing only fungal sequences. One of the major fungal clades contains all known TPPs whereas the other contains characterized endosedolisins. We identified four Cluster Specific Inserts (CSIs) in endosedolisins, of which CSIs 1, 3 and 4 appear as solvent exposed according to structure modeling. Part of CSI2 is exposed but a short stretch forms a novel and partially buried α-helix that induces a conformational change near the binding pocket. We also identified a total of 15 specificity determining positions (SDPs) of which five, identified in two independent analyses, form highly connected SDP sub-networks. Modeling of virtual mutants suggests a key role for the W307A or F307A substitution. The remaining four key SDPs physically interact at the interface of the catalytic domain and the enzyme’s prosegment. Modeling of virtual mutants suggests these SDPs are indeed required to compensate the conformational change induced by CSI2 and the A307. One of the two small fungal clades concerns a subfamily that contains 213 sequences, is mostly similar to the major TPP subfamily but differs, interestingly, in position 307, showing mostly isoleucine and threonine.

**Conclusions:**

Analysis confirms there are at least two sedolisin subfamilies in fungi: TPPs and endopeptidases, and suggests a third subfamily with unknown characteristics. Sequence and functional diversification was centered around buried SDP307 and resulted in a conformational change of the pocket. Mutual Information network analysis forms a useful instrument in the corroboration of predicted SDPs.

**Electronic supplementary material:**

The online version of this article (10.1186/s12859-018-2404-y) contains supplementary material, which is available to authorized users.

## Background

### Proteases are ubiquitous enzymes that can be classified in many ways

Proteases or peptidases degrade proteins by hydrolysis of peptide bonds. They are involved in various biological processes such as cell death [1], nutrition [2] and infections [3]. MEROPS [4], the peptidase database, classifies proteases based on the catalytic mechanism into the types of asparagine, aspartic, cysteine, glutamic, metallo, serine and threonine proteases. Further hierarchical classification into clans and families is based on homology and structure similarity. The remainder of the proteases fall into five clans of mixed catalytic type, clans that are further organized in homologous families, and a class of proteases with unknown catalytic mechanism. Proteases can also be classified based on other characteristics. A major difference can be made between endo- and exopeptidases, where the latter include aminopeptidases, carboxypeptidases, dipeptitidyl-peptidases and tripeptidyl-peptidases (TPP) as well as dipeptidases and peptidyl-dipeptidases.

### Sedolisins are acid proteases related with the basic subtilisins

Serine proteases are proteases in which a serine serves as the nucleophylic amino acid in the catalytic site. The catalytic site is most often formed by a triad which can differ among the different unrelated superfamilies. Currently 12 clans or superfamilies with 55 families have been assigned by MEROPS [5]. One of the most important clans is the SB clan that contains the common subtilisins (S8), which include the kexins [6], and the rather rare sedolisins (S53, for review see [7]). Sedolisins have been described in prokaryotes and eukaryotes. Interestingly prokaryotic and eukaryotic sedolisins are very distant showing often less than 25% sequence similarity. Despite a large difference in optimal activity pH, there is ample evidence the two subfamilies form a superfamily. Note that superfamilies can be hierarchically organized into many different subfamilies with many different, sometimes unknown, functional characteristics. In 2001 the first structure of a sedolisin, endosedolisin PSCP from *Pseudomonas* sp. 101*,* complexed with inhibitor iodotyrostatin, was resolved (PDB code 1GA4) [8], shortly followed by a structure from kumamolysin from *Bacillus sp. MN-32* (PDB codes 1T1E for precursor and 1GT9 for mature peptidase [9]). Although sequence similarity between sedolisins and subtilisins is low, structural alignments clearly indicate they are homologous [10] since they have similar folds. The basic subtilisins have a triad that consists of the serine, a histidine and an aspartate, the acid sedolisins have a homologous serin, a homologous glutamate that replaces the histidine as well as a non-homologous aspartate [11]. Also the oxyanion aspartate appears as homologous. Sedolisins also have a calcium binding site albeit at a different position than subtilisins [10].

The best studied sedolisin is human lysosomal CLN2 since mutant forms are involved in the fatal classical late-infantile neuronal ceroid lipofuscinosis or Batten disease. The structure (PDB codes 3EDY for precursor and 3EE6 for mature peptidase) of this tripeptidyl aminopeptidase has been determined and a number of publications describe the effect of many mutations found [12–14]. Of particular interest is W542 which has been shown to be required for activity. The W542L mutant was shown to be retained in the ER which suggests misfolding [15]. In addition, W290L and W307L showed largely reduced activities.

### Sedolisins have a large prosegment that appears to have various functions

The processing of subtilisins and sedolisins is similar. Both have a large and similar prosegment that appears to be able to form an independent domain that seems to be involved in correct folding of the core or the catalytic domain [16, 17]. The prosegment and catalytic domain are separated by a short propeptide or linker that is removed during zymogen activation at low pH. In general it has been shown that prosegments assist in refolding as well as targeting (for review see [18]). For human TPP it has been shown that prosegment and catalytic domains have multiple molecular interactions including salt bridges and hydrogen bonds, covering 15% of the solvent accessible surface of the catalytic domain [19]. It has been shown that the prosegment of human TPP also functions as an inhibitor [16]. Secretome analysis of for instance *Botrytis cinerea* has shown certain paralogs consist of the core part of the enzyme only [20].

### Fungal sedolisins can have endo- or tripeptidyl-peptidase activity

Other characterized eukaryotic sedolisins are of fungal origin. Scytalidolisin [21], grifolisin [22] and aorsin were the first fungal enzymes characterized as sedolisins. More recently, four homologs from *Aspergillus fumigatus* were characterized. SED_A was, similarly to aorsin form *Aspergillus oryzae,* characterized as an endosedolisin, whereas SED_B, SED_C and SED_D were shown to have TPP activity [23]. Endo and TPP activity have been described for subtilisins. The authors suggested furthermore endosedolisins cluster in a different clade than TPPs, suggesting that gene duplication has resulted in functional diversification. A recent genome paper of fungal plant pathogens *B. cinerea* and *Sclerotinia sclerotiorum* showed that acid secreting fungi such as phytopathogens *B. cinerea, S. sclerotiorum* but also the saprophytic *Aspergilli* show relatively few subtilisins and many sedolisins, as compared to non-acid secreting fungi such as *Giberella zeae* [24]. Interestingly, yeasts from Saccharomyces and Schizosaccharmyces completely lack homologs. This also suggests functional diversification has occurred. Here we study the functional redundancy and diversification of fungal sedolisins by computational analysis. We reconstructed a phylogenetic tree that, together with the underlying multiple sequence alignment (MSA), was used for the identification of cluster specific inserts (CSIs) and specificity determining positions (SDPs), which form the sequence characteristics that can explain functional diversifications. Modeling of wild type and mutant sequences was performed in order to show how functional diversification into endosedolisins and TPP has likely occurred, demonstrating important roles for part of CSI2 and the position homologous to human TPP W307.

## Methods

### Sedolisin sequence identification

A first HMMER [25] profile, built from the MEROPS [5] holotype sequences from the MEROPS database, was used to search a database containing the complete proteomes of 56 fungi and 186 non fungal eukaryotes complemented with the PDB and Swissprot database, yielding 230 sequences of sedolisin homologs. A sequence hallmark scrutiny for the presence of catalytic site and oxyanion residues was performed using MEROPS Batch BLAST [26], followed by a structural scrutiny finally yielding a total of 204 high fidelity sequences. These were aligned using MAFFT’s [27] iterative refinement method and the resulting MSA was manually corrected using as criteria that secondary structure elements (taking as reference 3EE6) should be represented by each sequence, combined with entropy minimization. The resulting MSA was used to construct a preliminary maximum likelihood tree using PHYML. The preliminary tree has three clearly separated, major clades and the three corresponding sub-MSAs were used to construct subfamlily specific HMMER profiles. These were used to iteratively screen HMMER’s Reference Proteomes dataset restricted to eukaryotes using the procedure as described by the recent superfamily classification software HMMERCTTER [28] resulting in a total of 2203 sequences.

### MSA and phylogeny

**Fig. 1:**
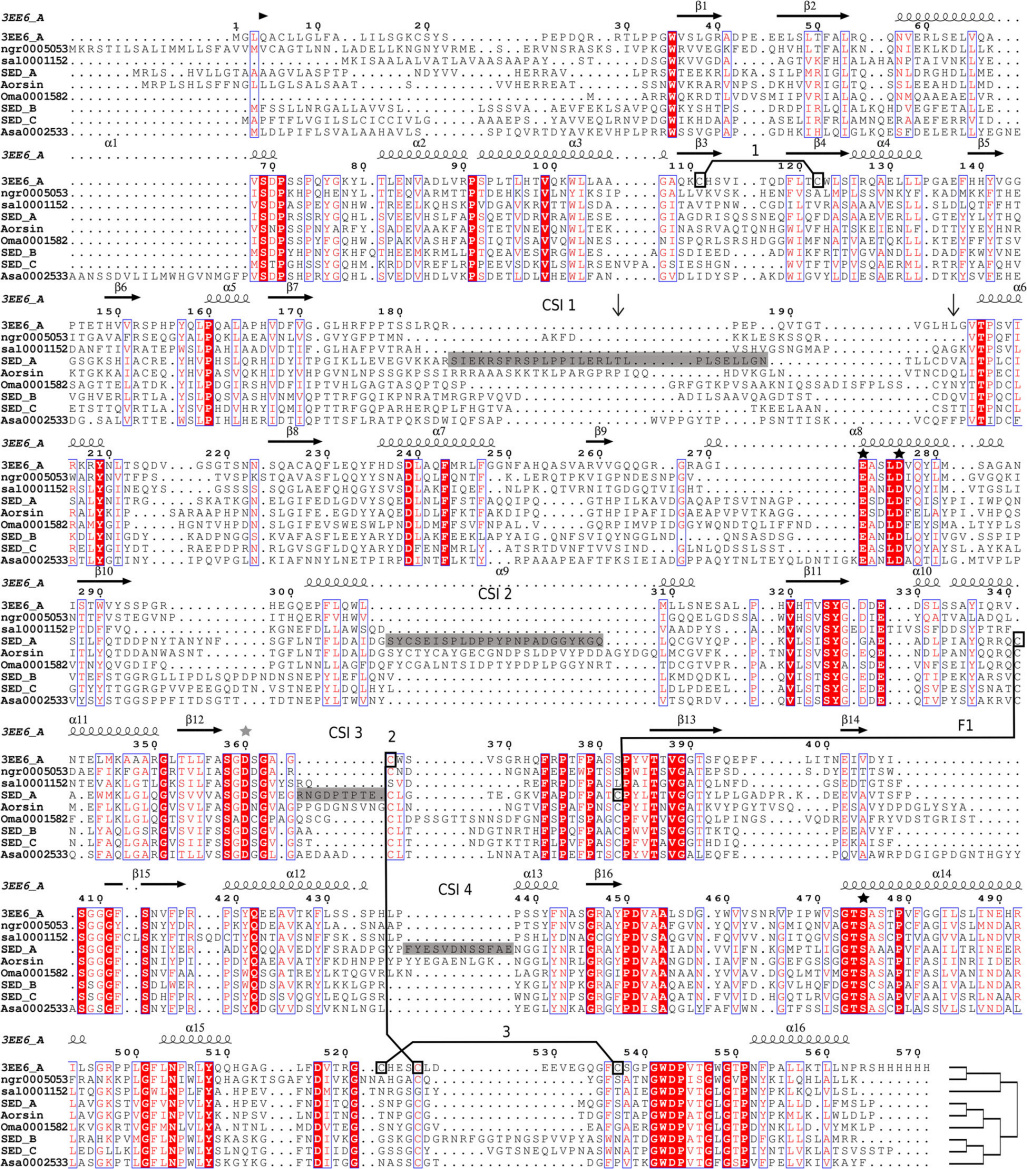
Excerpt of Eukaryotic Sedolisin Sequence Alignment. The demonstrated sequences are representatives of the three mayor phylogenetic clusters, as indicated by the tree placed at the end of the alignment, extracted from the complete MSA (Additional file 5). 3EE6, ngr0005053 and sal0001152 are non-fungal sequences; SED_A and Aorsin are fungal endosedolisins; SED_B and SED_C are fungal TPPs. Oma0001582 and Asa0002533 are additional fungal sequences with unknown characteristics. Horizontal arrows and helices indicate sheet and helix regions, respectively. Nomenclature of secondary structure elements and numbers according to 3EE6. The vertical arrows represent beginning and end of the peptide linker. Sequence in gray box represents the SED_A inserts (numbers according to SED_A sequence): CSI1 (190–217), CSI2 (345–371), CSI3 (427–435) and CSI4 (513–525). Black stars indicate catalytic residues E, D, and S whereas the gray star indicates the oxyanion D. Connected boxes, numbered 1, 2 and 3 indicate disulfide bridges identified in the 3EE6 structure. F1 points to two cysteines strictly conserved among all fungal sedolisins but absent in non-fungal sedolisins. Red shading (identity) and fonts (similarity) highlight conserved positions

The final MSA was constructed using MAFFT’s [27] -add GINSI with iterative refinement using the 204 sequence MSA as seed, its representation (Fig. 1) was made using Endscript [29, 30]. The MSA was trimmed using BMGE [31] (−g 0.3 and h 0.8) which resulted in a trimmed MSA that maintains more than 90% of the columns corresponding to the β-sheets. Maximum likelihood phylogeny was constructed using PHYML3.1 [32] using the WAG model of amino acid substitution and a discrete gamma model of four categories and a shape parameter of 1.4, as determined by prior ProtTest [33] analysis. For statistical support we used FastTree [34] with 1000 bootstraps using the resources available at http://booster.c3bi.pasteur.fr./new/. Graphical representations were made using iTOL [35].

### Identification of specificity determining positions (SDPs)

We identified Cluster Determining Positions (CDPs) using SDPfox [36]. MISTIC [37] was used to determine levels of Mutual Information (MI) between positions or columns of the MSA. Initially, CDPs are accepted as SDP when they contain at least two direct connections with other CDPs, using MISTIC’s default z-score cut-off of 6.5. CDPs with a single direct connection are considered as putative SDPs (pSDP) and require additional evidence in order to become accepted as SDP. Cytoscape [38] was used to identify and draw sub-networks of directly connected SDPs. Sequence logo’s were made using Weblogo [39].

### Structure analysis

Tertiary structures of sedolisins were obtained from the Protein Data Bank [40]. 3EE6 [19], corresponding to mature human TPP was used as reference. Models were made using I-Tasser [41] using either default settings or using 3EE6 Chain A as the reference model. The SED_A dimer was made by structural alignment of the SED_A monomer model to both the 3EE6 A and B chains. Visualization was performed using VMD [42] which included structural alignment using the STAMP [43] extension. The pocket predictions for 3EE6 and the SED_A and SED_B models were performed with the software Fpocket [44] using the default parameters.

## Results

### Datamining, multiple sequence alignment and phylogeny

In order to perform structure-function analysis of eukaryote sedolisins we set out to obtain a representative collection of sequences, while trying to avoid the inclusion of sequences corresponding to pseudogenes or derived from incorrect gene models. High sensitivity was obtained by applying HMMER iteratively, whereas specificity was obtained by an initial sequence scrutiny and using strict cut-off thresholds using a HMMERCTTER [28] procedure, for details see materials and methods. A total of 2203 sequences were aligned and an excerpt of the final MSA is shown in Fig. 1. In general, eukaryotic sedolisins are largely conserved, including the prosegment part. Interestingly, of the three disulfide bridges identified in the resolved structure 3EE6, only the second appears to be conserved among eukaryotes. A trimmed MSA, lacking low quality sub-alignments, was used to reconstruct a maximum likelihood tree using FastTree 2 [34] with 1000 bootstraps.

**Fig. 2:**
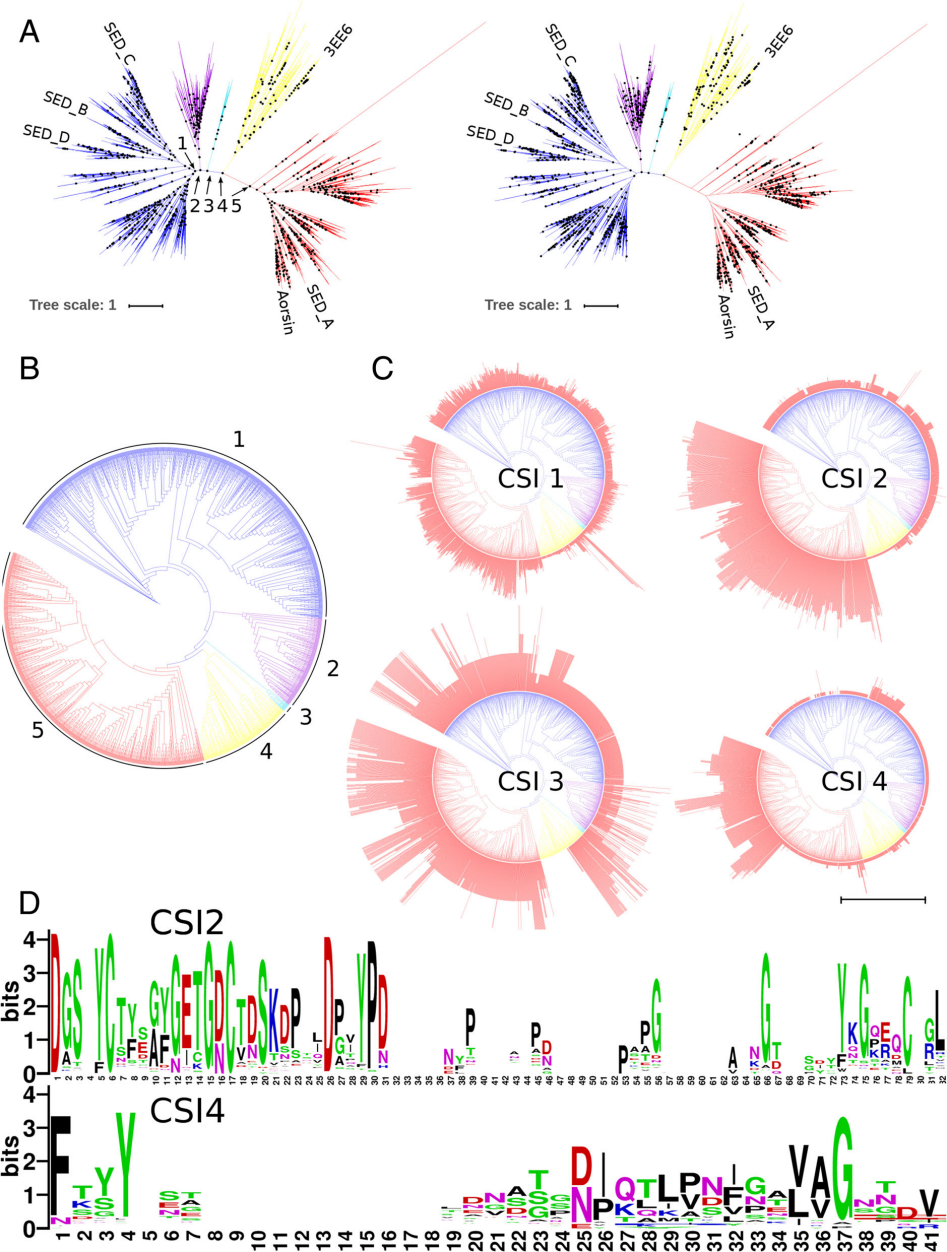
Phylogenetic Clustering and Cluster Specific Inserts of Eukaryotic sedolisin. Clades: Blue: Fungal Hypo-TPP; Purple: Fungal Uncharacterized 1; Cyan: Fungal Uncharacterized 2; Yellow: Non-Fungal; Red: Fungal Hypo-Endo. **a** Midpoint rooted radial phylograms showing normalized (Left, > 0.95) and Felsenstein (Right, > 0.66) bootstrap support. The scale bar indicates 1 amino acid substitution per site. **b** Circular cladogram. **c** Circular cladograms showing the clustering of the four CSIs: the length of each sequence’s CSI is represented by a red bar. The scale bar at the bottom corresponds with 25 amino acid residues. **d** Sequence logos of the CSI2 and CSI4 regions of the hypo-endosedolisin cluster

The tree (Fig. 2a) shows five major clades of which four contain only fungal sequences and one contains only non-fungal sequences. A similar tree was obtained when reconstruction was performed with PHYML (See Additional file 1). The apparent random taxonomic distribution of the sequences over the two well separated, major fungal clades indicates the bifurcation is caused by a functional diversification. Also the larger minor fungal clade has at least both asco- and basidiomycete sequences. In correspondence with Reichard and coworkers [23], one of the major fungal clades, containing 785 sequences, contains SED_A from *A. fumigatus* and Aorsin [45] from *A. oryzae* that have both been characterized as endopeptidases. The other major fungal clade, with 971 sequences, contains SED_B, SED_C and SED_D from *A. fumigatus,* all characterized as TPPs [23]. Although biochemical evidence is scarce, the above mentioned data suggest sequence diversification has resulted into endo- and TPP sedolisins, hence for the remainder of the manuscript we will refer to the sequences and these major fungal clades as Hypo-endosedolisin (or Hypo-Endo) and Hypo-TPP. Both endo and TPP activity has been shown for non-fungal sedolisins, hence, as such we have no indication regarding the state of the ancestral enzyme. In addition, at least human TPP has been shown to have endo activity at low pH [46]. The other fungal clades, with 213 and 18 sequences respectively, do not contain any characterized sequence.

### Cluster specific inserts

The MSA demonstrates the presence of four Cluster Specific Inserts (CSIs) we identified in the Hypo-endosedolisins. Fig. 2c shows the distribution of insert length on the phylogeny thereby demonstrating an intricate clustering pattern. CSI2 has about 25 amino acids and is present in all Hypo-endosedolisins whereas CSI4 has about 40 amino acids and is found only in the large subclade of the Hypo-endosedolisin clade. Both show moderate levels of conservation (Fig. 2d). CSI3 is present in all Hypo-endosedolisins as well as in certain Hypo-TPPs and some non-fungal sedolisins. CSI1 is present in all sedolisins but is longer in the Hypo-endosedolisins. CSIs 1 and 3 show no clear conservation.

**Fig. 3:**
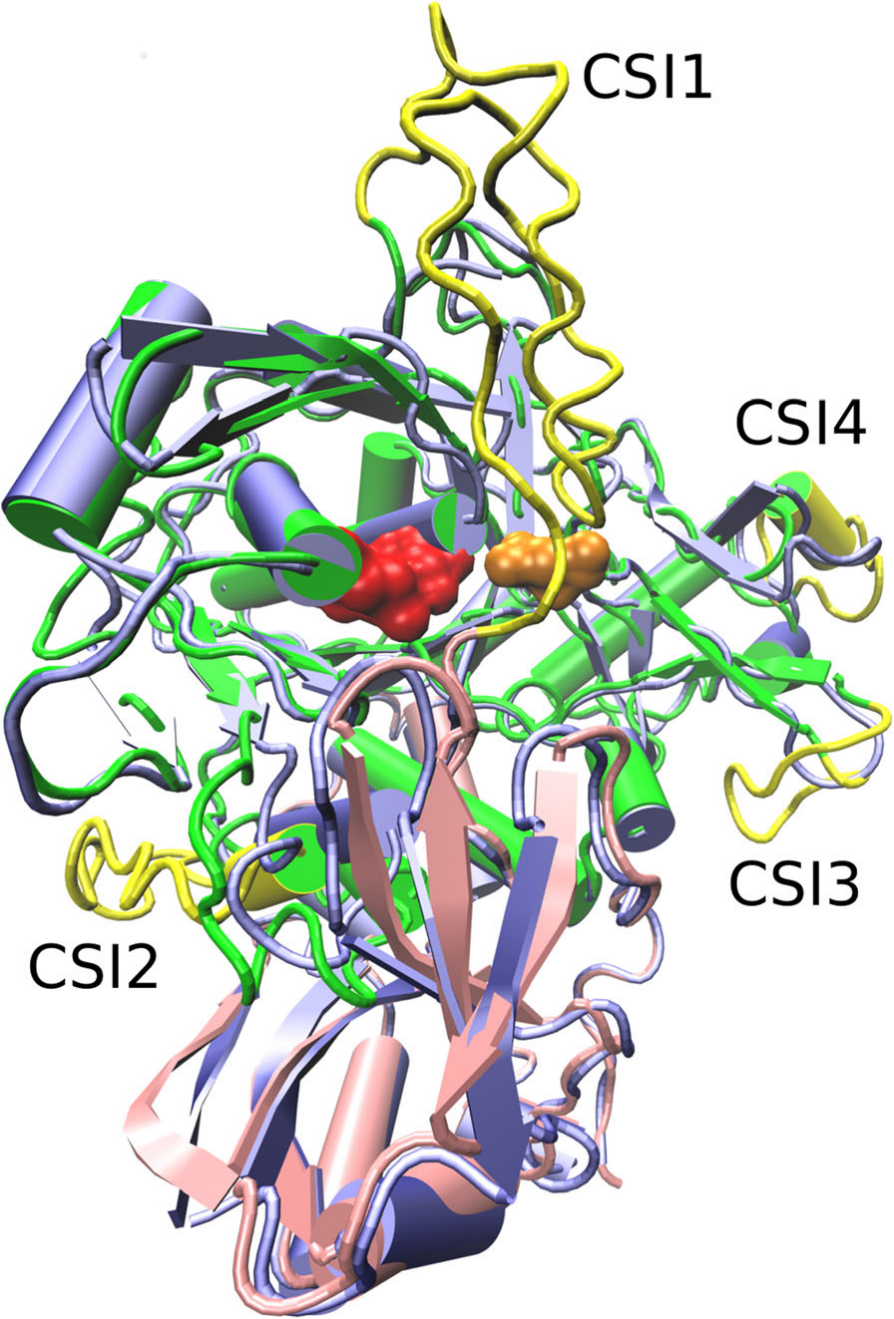
The Cluster Specific Inserts are Solvent exposed. Structural alignment of the 3EE6 structure, represented in blue cartoon, and the SED_A model represented in pink (prosegment) and green cartoon (core) with CSI1 to 4 in yellow cartoon. The red and the orange spheres represent the catalytic residues and oxyanion respectively

Because structure determines function we wondered if the CSIs would interfere in the core structure of the protein or if they might appear as solvent exposed loops. We created a model of SED_A and structurally aligned it with 3EE6 (Fig. 3). Overall, the model is very similar to the 3EE6 structure and the non-homologous CSIs 1, 3 and 4 are indeed predicted at the surface of the mature protein. CSI2 is largely exposed but forms a partially buried helix as is discussed below. This confirms that the CSIs do not necessarily affect the basic functional fold of subtilisin-like proteases. Although in the absence of homologous template the prediction of the loop structures formed by the CSIs is not very reliable, the model does allow some general predictions. CSI1 is located next or into the prosegment and likely does not form part of the mature enzyme but rather forms part of the propeptide or linker region (See Fig. 1). CSI2 and CSI4 are located on opposite ends of the predicted binding pocket (Not shown). CSI3 appears near the calcium binding site. Last we checked if the CSIs might interfere with dimer formation, since human TPP occurs as a dimer. In the dimer model (Additional file 2a), CSI1 occupies part of the same space as CSI3 and CSI4, the model seemingly being incorrect. Since we suspect that CSI1 is part of the propeptide that is removed upon zymogen activation, we also made a dimer model where CSI1 is absent. Additional file 2b shows CSI2, 3 and 4 do not constitute any clear spatial conflict. Next we analyzed the structure of the 3EE6 dimer interface by means of PISA [47]. The three interfaces with the highest scores indicate the presence of Zn576, designated to Chain A, that interacts with HIS197 and ASP457 from chain A and with GLU529 from chain B (Additional file 2c). Similarly Zn577, designated to chain B, interacts with HIS197 and ASP457 from chain B and with GLU529 from chain A, thereby establishing the dimer interface. Interestingly, the linker region of 3EE6 corresponds with positions 181 to 196, This further supports that CSI1 forms part of the linker-peptide that is removed during maturation. Finally, we checked if CSI1 showed high mutual information with either CSI3 or CSI4 (Additional file 2d), which was not found.

**Fig. 4:**
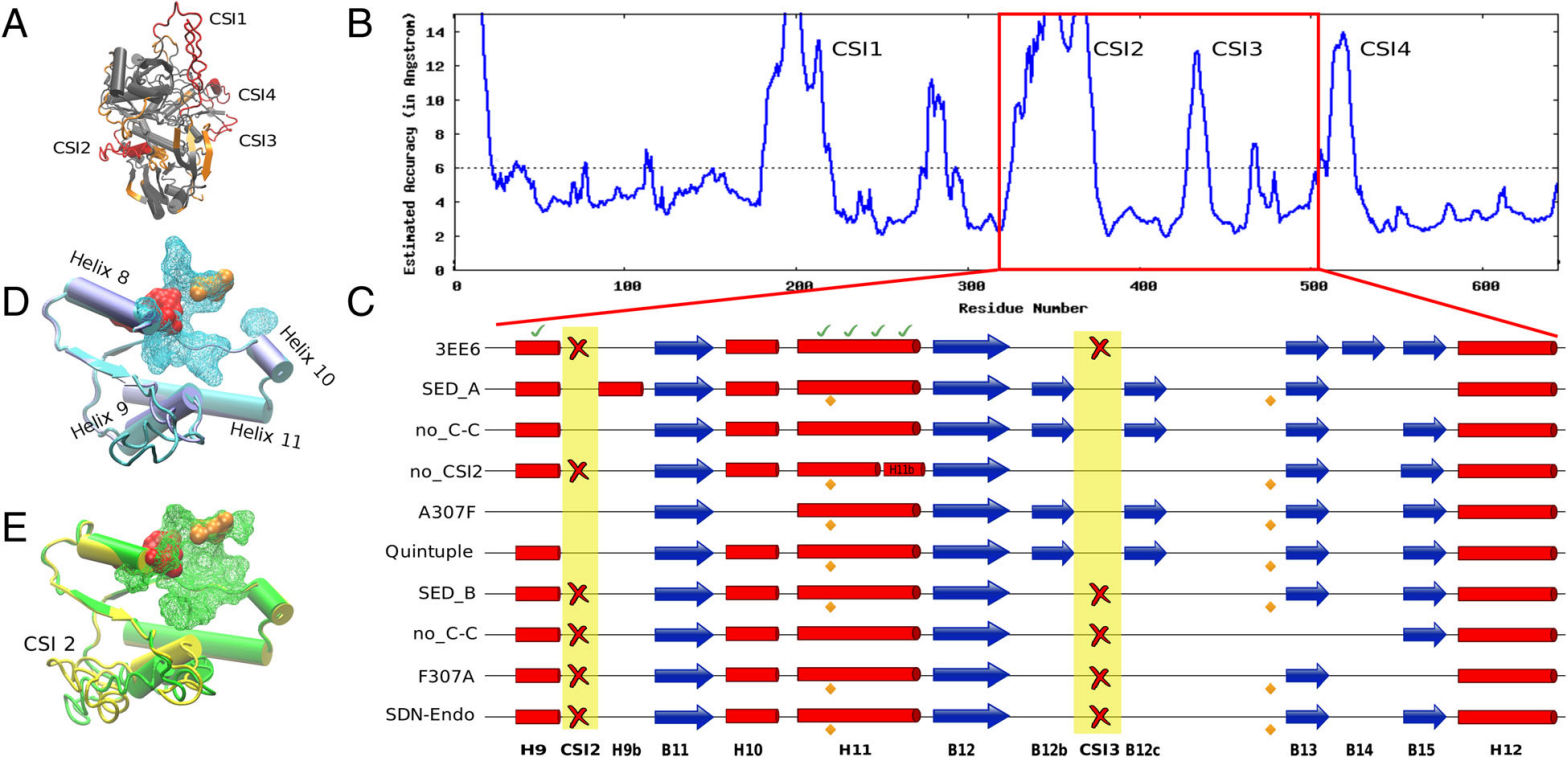
Conformational Differences among Wild Type and Mutant Sedolisins. **a** Cartoon of the SED_A model with regions that have a resQ score below 5 Å in grey, homologous regions above 5 Å in orange and CSIs with resQ above 5 Å in red. **b** ResQ plot of SED_A modeling. **c** Schematic representation of the major structural differences in secondary structure elements of the polypeptide between helices 9 and 12 observed in WT and mutant models of SED_A and SED_B as compared to the 3EE6 structure. Quintuple refers to the virtual SED_A A343F-F92L-E404L-K407Q-L410S mutant. Yellow diamonds indicate conserved cysteines that are possibly involved in a novel disulfide bridge. Approximate positions of SDPs 307, 343, 346 and 349 are indicated from left to right by a green check mark. Absent counterparts are represented as a red cross. Helices are in red cyinders and sheets in blue arrows. **d** Local detail of the structural alignment of 3EE6 (blue cartoon) with model obtained for WT SED_B (cyan cartoon). The wireframe indicates the predicted binding cleft with the catalytic triade in red and the oxyanion in orange. **e** Local detail of the structural alignment of WT SED_A model (green cartoon) and the SED_A quintuple mutant model (yellow cartoon). The wireframe indicates the predicted binding cleft with the catalytic triade in red and the oxyanion in orange. Helix numbers are indicated in the 3EE6 structure

Figure 4 shows the conformational differences between 3EE6 and the wild type (WT) models SED_A and SED_B as well as a number of mutations that are discussed in the SDP section further below. An important conformational difference is the additional α-helix found in the SED_A model, referred to as H9b, that partially originates from CSI2 (See Fig. 4e). As a result the location of Helix 9 also slightly differs. This seems to have an effect on the binding pocket, as can be seen by a comparison of predicted pockets of SED_B (Fig. 4d) and SED_A (Fig. 4e). The virtual SED_A mutant lacking CSI2 does not show helix 9b and retains a structure similar to 3EE6.

### SDP identification

We used SDPfox [36] to identify CDPs, positions that contribute significantly to the underlying clustering. We identified 26 CDPs between the Hypo-endo and the Hypo-TPP clusters. Then we performed an analysis of mutual information between positions using MISTIC [37]. Mutual information expresses levels of covariation and high levels suggest co-evolution. CDPs might result form genetic drift but CDPs that show high levels of interaction are more likely SDPs. We envisage that the functional characteristics of phylogenetically well separated subfamilies, such as the Hypo-endo and Hypo-TPP sedolisins, are the result of the interaction of multiple positions that have somehow co-evolved. As such, possibly one or more sub-networks of directly connected CDPs exist. We consider all CDPs that connect directly to at least two other CDPs with a score higher than MISTIC’s default threshold of 6.5 as SDP. CDPs with a single connection are initially considered as pSDP. Eventual sub-networks of directly connected SDPs are considered Specificity Determining Networks (SDNs) that not only substantiate that CDPs are SDPs but also show which positions have co-evolved towards a certain diversification.

**Fig. 5:**
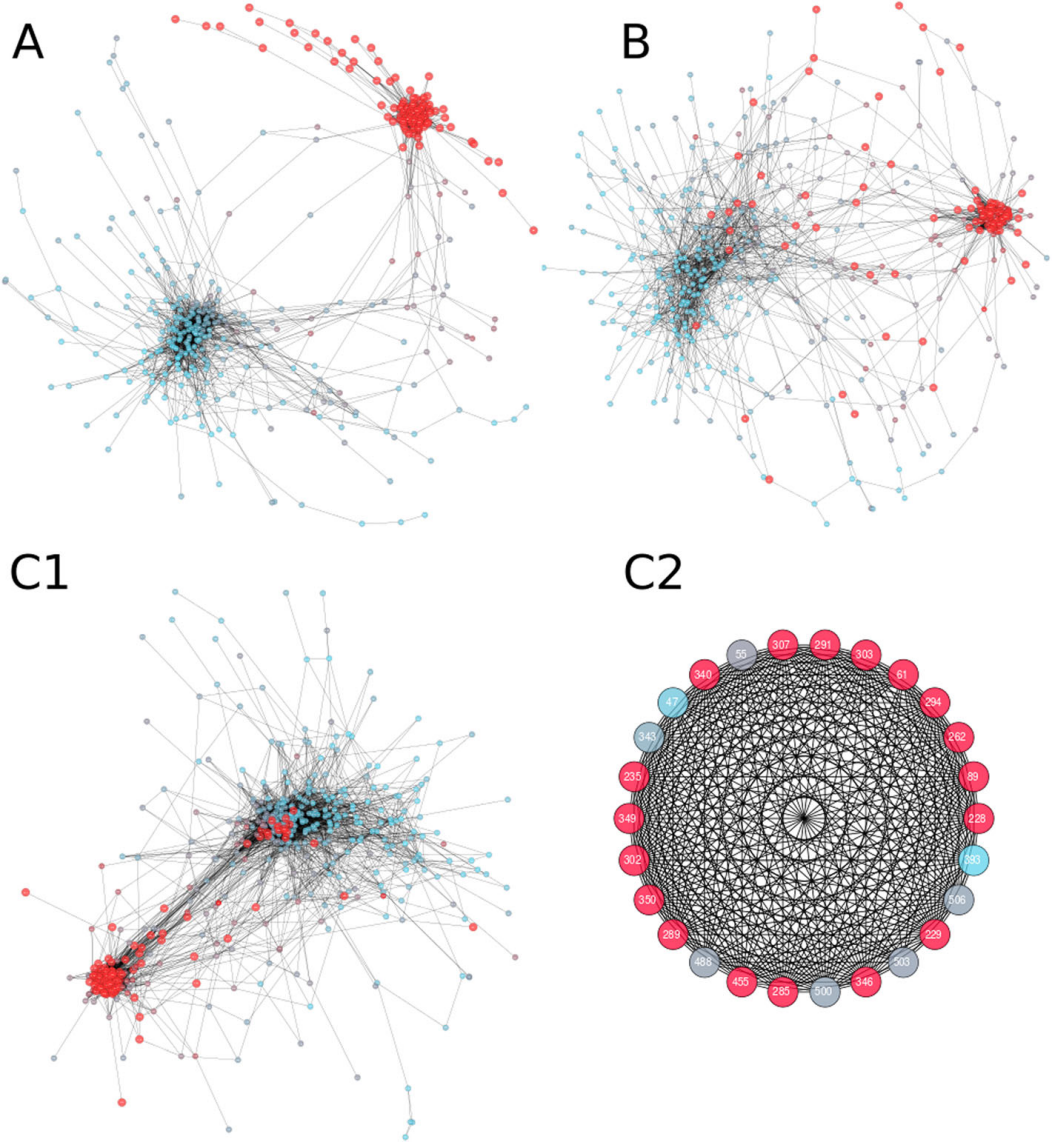
Cluster dependent Mutual Information Networks. Shown are the MI networks obtained for the Hypo-Endo cluster **a**; the Hypo-TPP cluster **b**; and the combined Hypo-Endo / Hypo-TPP dataset **C1**. Red nodes represent the same positions in the three MI networks demonstrating topological similarity between the three networks. (**C2**) CDP sub-network obtained using MISTIC’s default threshold

From a theoretic point of view we must assume that the diversification process in a certain clade has been independent from that in another clade. On the other hand we can also envisage that the diversification processes, although strictly independent, might affect the same positions. Since, as we stated before, we do not know the functional characteristics of the common ancestor, we can also not know in which of the clades a diversification has occurred. As a result, a priori one does not know which dataset should be used to determine MI levels. We first performed a global MI analysis comparing the sub-networks obtained when using 1) only the Hypo-endo; 2) only the Hypo-TPP; and 3) the Hypo-endo and Hypo-TPP sequences combined. Fig. 5 shows that when separate clades are used as dataset, similar networks with two dense clusters are identified. Also when sequences are combined, two dense clusters are found but these are more heavily connected. As such, it seems that, in general, the diversification processes that have occurred in the two clades, concern similar positions. Next we plotted the 26 identified CDPs and the sub-network they form upon MISTIC analysis of the combined sequence set. Fig. 5C2 shows that, when the default threshold of 6.5 is applied, all CDPs connect, seemingly, to all CDPs. The default threshold of MISTIC, a z-score of 6.5, is set for sequence sets with 400 sequence clusters, the combined sequence set showed 533 clusters. Hence, in order to correctly identify which connections are significant, the threshold should be higher. However, since there is no easy method to determine the MI threshold, we plotted the sub-networks that result from the smaller Hypo-Endo and Hypo-TPP datasets, which had 214 and 300 clusters each.

**Fig. 6:**
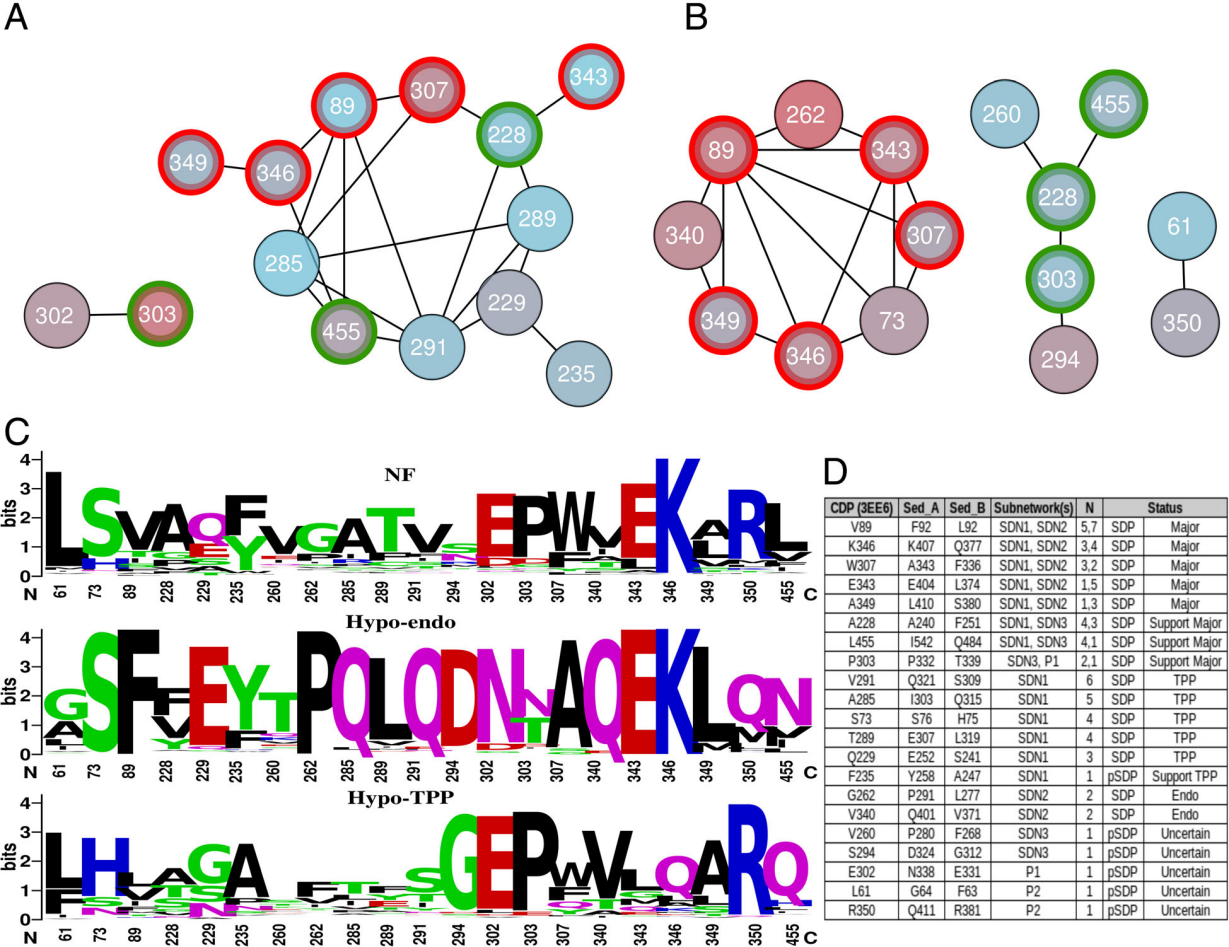
Specificity Determining Positions and Networks. SDPs, i.e. CDPs with high mutual information determined on the Hypo-TPP **a** and Hypo-Endo **b** subclade datasets. SDPs and pSDPs, as defined in the text, form sub-networks SDN1, SDN2 and SDN3 as well as two pairs of positions P1 and P2. **c** Sequence logos show residue conservation at the involved positions in the non-fungal (NF), Hypo-Endo and Hypo-TPP clades. **d** The table shows that various SDPs from sub-network SDN1 are present in either sub-network SDN2 (red encircled in **a** and **b**) or SDN3 (green encircled in **a** and **b**). N indicates the number of connections to other SDPs in the networks it takes part, respectively Numbers are according to 3EE6, except for the table where corresponding numbers of SED_A and SED_B are included

Figure 6 shows the obtained sub-networks and sequence logos of the identified SDPs and pSDPs in the non-fungal, the Hypo-Endo and the Hypo-TPP clades. The Hypo-TPP MI analysis results in large sub-network SDN1, with nine SDPs and three pSDPs, as well as a pair of connected pSDPs. The Hypo-Endo MI analysis results in large sub-network SDN2 with eight SDPs, small sub-network SDN3 with two SDPs and three pSDPs as well as a pair of connected pSDPs. Comparison of Hypo-TPP sub-network SDN1 on the one hand and Hypo-Endo sub-networks SDN2 and SDN3 on the other hand shows that diversification towards TPP and endosedolisin is governed by similar sets of positions (Fig. 6). SDPs 89, 307, 343, 346 and 349 all take part in SDN1 and SDN2. Furthermore, SDPs 228 and 455 take part in SDN1 and SDN3. This might point to a situation where there is actually a single set of SDPs that might combine into a single sub-network, which might be obscured by a possibly too strict MI threshold. However, analysis of the crude MI data shows SDN2 and SDN3 only connect when a very low threshold is applied. Hence, it is more likely SDN2 and SDN3 concern different diversification processes.

### Structure-function analysis

**Fig. 7:**
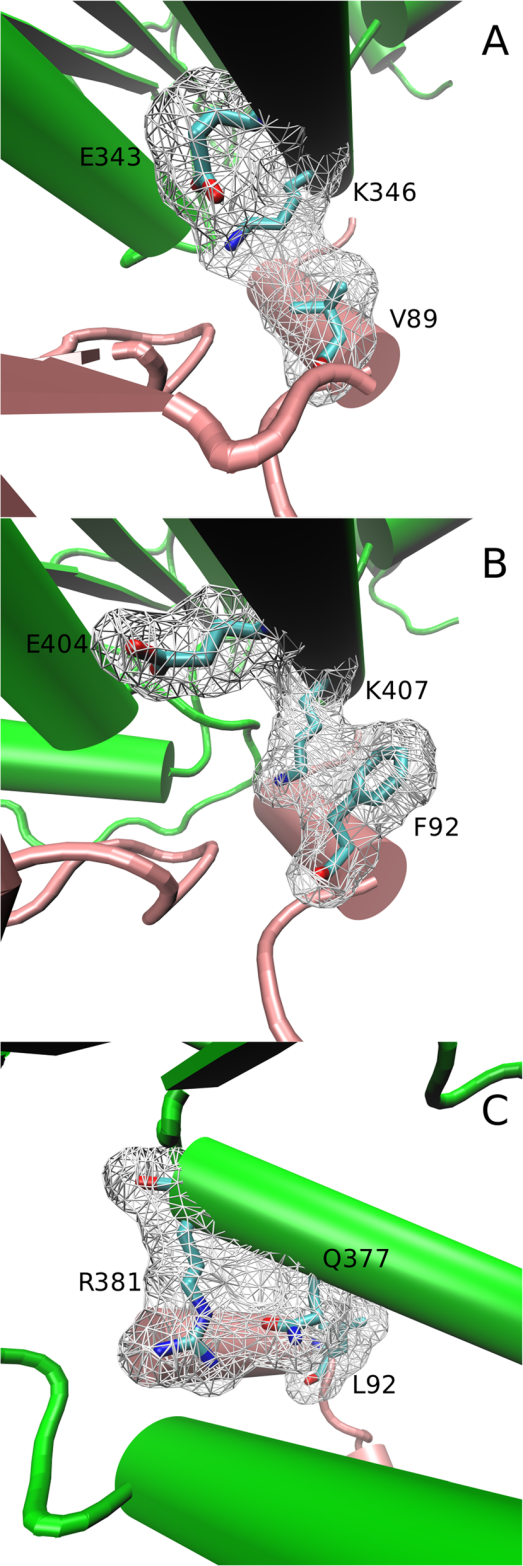
Interaction of SDP346, SDP343 and SDP89 at the interface between core and prosegment. Cartoons showing interaction in 3EE6 (**a**); SED_A (**b**); and SED_B (**c**). In 3EE6 K346 forms a salt bridge with E343. The different location of Helix 9 in SED_A appears to separate K407 and E404 which is compensated by F92 from the prosegment. In the SED_B model Q377 is folded internally and interacts with R381. Green cartoon corresponds with core whereas pink cartoon represents prosegment. The SDPs are represented in licorice element, while surf represents their 5 Å radius

First we checked if any of the SDPs or CDPs might interact with the catalytic site or form part of the binding cleft. All SDPs and CDPs, except CDP229 locate at over 5 Å from any of the catalytic residues (Additional file 3). Probably not all SDPs of the identified SDNs will have the same impact on enzyme function. The comparison of sub-networks obtained by the two independent analyses indicate SDPs 89, 307, 343, 346 and 349 are the most likely to be involved in enzyme specificity since they take part in both SDN1 and SDN2, sub-networks that show a higher level of connectivity than SDN3. These we consider key SDPs. The most highly connected SDPs with a large physicochemical difference between the two clades, are most likely to explain the functional diversification. Both SDP346 and SDP89, located in the core and the prosegment respectively, are highly connected to other SDPs in both sub-networks (Fig. 6d). Core SDP346 shows a substitution of the amphipathic, positively charged lysine in Hypo-Endo (K407 in SED_A) to most often the polar glutamine in Hypo-TPP (Q377). In human TPP 3EE6, K346 is located on the interface between core and prosegment interacting with E343 (Fig. 7a). Although SED_A has the same residues, K407 (homologous to K346 from 3EE6, see Fig. 6) physically interacts with SDP89 (F92). This, according to the model, seems to be caused by a slight dislocation of helix 9 in SED_A, in its turn forced by the additional helix 9b, which results from CSI2. The corresponding Q377 of SED_B was modeled internally and is likely stabilized in the opposite direction by the polar residue R381 in SED_B (Fig. 7c) whereas F92 is replaced by leucine. The hydrophobic interaction between the large F92 and the aliphatic chain of K407 suggests SDP346 is involved in the interaction between prosegment and core. The ε-amino group of K407, no longer forming a salt bridge with E404 can be envisaged to be protonated upon secretion into the acid environment, thereby destabilizing the interaction between core and prosegment regions. Given the suggested role of the prosegment in sedolisin folding and stabilization [16] this could explain at least part of the diversification.

SDP349 also occurs in the vicinity of SDP346 and connects directly to SDP346 (SDN1 and SDN2) and SDP89 (SDN2). Alanine, common in Hypo-TPP is slightly less hydrophobic than the predominant leucine of Hypo-endosedolisins. SDP340 is also in close range of SDP346 and connects directly to SDP89 in SDN2. It shows predominantly a polar glutamine in the Hypo-Endo and a hydrophobic valine in the Hypo-TPP clade. Interestingly, SDP340 is found next to position 341 that corresponds with one of two cysteines that are absent in non-fungal sedolisins and strictly conserved in fungal sedolisins (See Fig. 1). Since strictly conserved cysteine pairs often correspond with disulfide bridges we checked their orientation in the models of SED_A and SED_B. Although in both the SED_A (Additional file 4) and the SED_B model they are modeled at positions that seem to favor a disulfide bridge, this is not modeled. Nevertheless, the virtual C402A / C452A double mutant of SED_A also reverts to the structure lacking helix 9b (Fig. 4c). All together, this suggests that both SDN1 and SDN2 are related to the predicted structural changes discussed in the previous section.

SDP307, part of helix 9, is a position that, according to the MI analysis, interacts with SDP89 in both SDN1 and SDN2, as well as with SDP346 in SDN2. Although in the structure of human TPP 3EE6 W307 is located at over 9 Å from the local SDP network described above, in the model of SED_A, its counterpart alanine is found at 3.7 Å (See Fig. 7b). W307 is buried in 3EE6 and present in most other non-fungal sequences and represented by an aromatic residue in the Hypo-TPP clade. Substitution of a buried aromatic residue by the small alanine will most likely result in a conformational change. We envisaged that the substitution might be related to the conformational changes identified between 3EE6 and SED_B on the one hand, and SED_A on the other. We made structural models of virtual mutants, exchanging SED_A for SED_B residues. The model of virtual mutant A307F in SED_A suggests the loss of helices 9, 9b and 10. Compensation should, according to the above train of thought, come from SDPs 89, 343, 346 and 349, which directly connect to SDP307. Hence, we modeled the quintuple A343F-F92L-E404L-K407Q-L410S SED_A mutant. The obtained model resembles 3EE6 and SED_B since the principal helices H9 and 10 are modeled nearly identically (Fig. 4d and e).

The other SDPs show a lower level of connectivity and might involve secondary compensations. In SDN2, SDP73 connects to all key SDPs except SDP349. SDP73 is mostly S in the Hypo-Endo and H/N in the Hypo-TPP clade. Structural analysis reveals that this SDP is located in the hinge region preceding the helix that contains SDP89. SDP262, connects to SDP89 and SDP343 and is a hydrophobic residue in Hypo-TPP and a P in Hypo-Endo. Its position does not indicate an important role in structure (i.e. the P does not induce a turn). SDP340 connects toSDP89 and SDP349 and is a Q in Hypo-Endo, whereas mostly V in Hypo-TPP, which, combined with its closeness suggests this is yet another mutation that has co-evolved in order to further compensate changes in the folding induced by the key SDPs and CSI2. SDP 285 from SDN1 seems another important position, as it connects to many SDPs among which SDP89 and SDP307. In Hypo-TPP there seems to be a low level of prevalence whereas in Hypo-Endo it is predominantly a Q. Also the remainder of the SDPs and pSDPs also possibly fulfill additional supporting roles in Hypo-Endo rather than Hypo-TPP, given their high conservation in Hypo-Endo only.

**Fig. 8:**
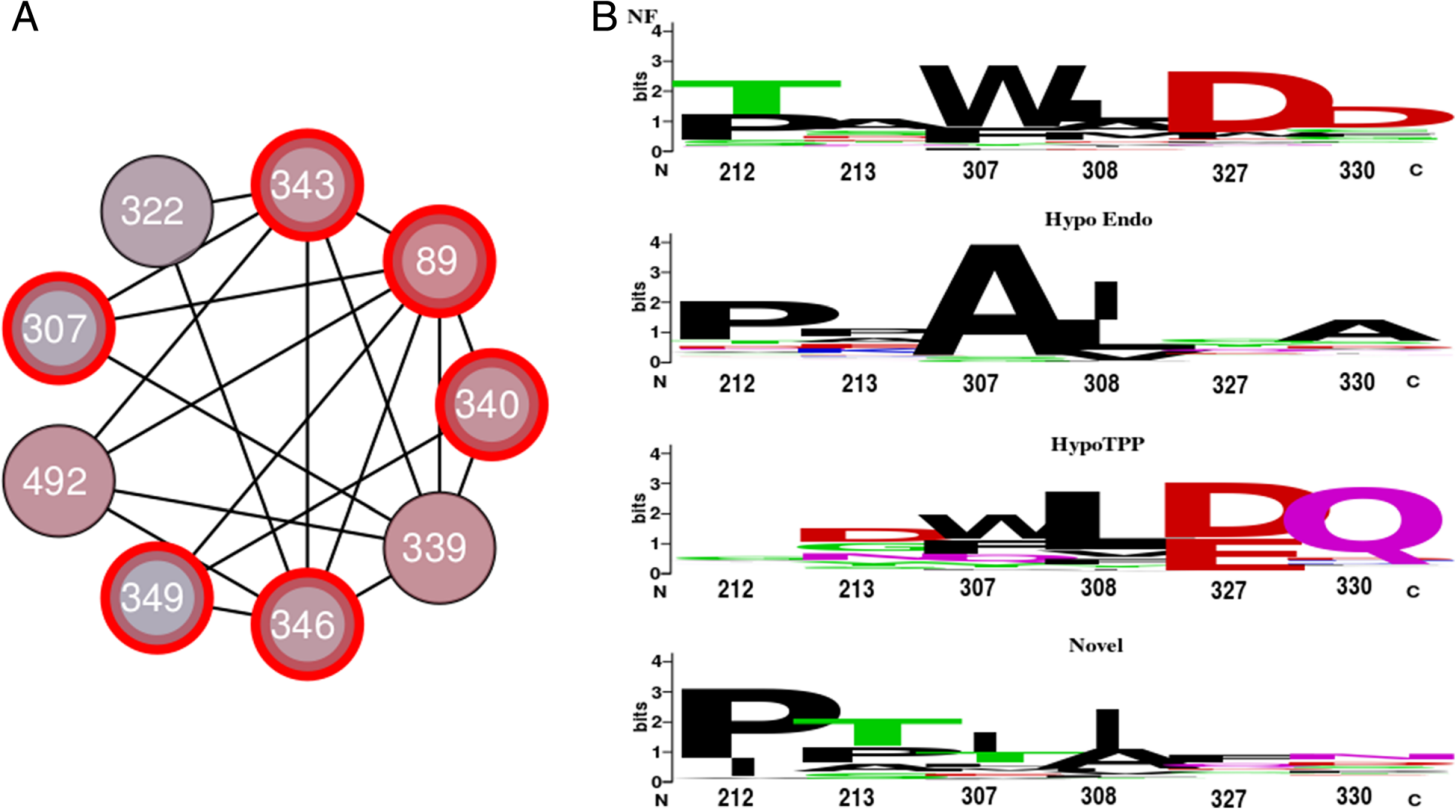
Structure-Function Prediction of the Novel Uncharacterized Sedolisin Subfamily. **a** MI sub-network of SDPs when the Hypo-Endo clade is compared to the clade with 213 novel fungal sedolisin sequences. Red encircled SDPs are shared with SDN2 of Fig. 6, showing SDPs involved in the diversification of the Hypo-Endo clade and indicating the novel clade is more similar to Hypo_TPP. **b** Sequence logos of CDPs identified in the comparison of the novel fungal sedolisin clade with the Hypo_TPP clade. Note that key CDP307 differs from non-fungal, Hypo-Endo and Hypo-TPP sequences. Numbers are according to 3EE6

Next we looked at the two smaller clades. The smallest has only 18 sequences which makes analysis troublesome. The other has 213 sequences and shows a proper statistical support (See Fig. 2a). Since none of its sequences has been characterized, structure-function analysis of this subfamily is only predictive in nature. We compared the clade with both the Hypo-Endo and the Hypo-TPP clade. CDPs identified in the comparison with the Hypo-Endo clade appear largely identical to those identified in the Hypo-Endo / Hypo-TPP comparison. Also when analyzed with MISTIC, using the Hypo-Endo dataset the sub-network is similar to SDN2, and contains all five key SDPs as well as SDP340 (Fig. 8a). This suggests the clade with uncharacterized sequences is similar to the Hypo-TPP subfamily. In order to show how it differs we analyzed the sequence logo of the SDPs identified when comparing the novel subfamily with the Hypo-TPP subfamily (Fig. 8b). Interestingly, the most obvious SDP is 307 that shows prevalence to I/T, rather then A or W/F in Hypo-Endo and Hypo-TPP respectively. Since clearly SDP307 forms a key residue in the specificity of sedolisins, we envisage this novel subfamily to have a yet to be determined enzyme characteristic.

## Discussion

We studied eukaryotic sedolisins by computational analysis of protein sequences. The protein sequences were obtained from a large set of EBI’s Complete Proteomes among which many are of fungal origin. Since it has been suggested that acidification by certain fungi is related to expansion of the sedolisin familiy in these fungi, the main attention of this study was directed at the evolutionary history of fungal sedolisins.

Sequences were aligned by MAFFT’s iterative refinement method and the resulting MSA was manually improved in order to correct poorly aligned residues, guided by hallmark residues and secondary structure conservation. Correction was likely required due to large taxonomic distances and the presence of CSIs which tend to disturb the alignment process. Prior to tree reconstruction, the MSA was subjected to trimming, guided by maintaining particularly β-sheets, in order to remove these CSIs and unreliably aligned subsequences. The clustering pattern of the CSIs largely corresponds with the phylogenetic clustering (See Fig. 2c), which confirms the topology of the tree is basically correct, as is also indicated by bootstrap support (See Fig. 2a). The Hypo-TPP clade shows a Felsenstein bootstrap support of 0.66, which is considered low. Felsenstein bootstrap yields a clear underestimation of statistical support: when any of the 971 leaves is differently placed, it results in a rejection of support. The recently proposed normalized bootstrap is over 0.95, strongly supporting this topology [48]. In addition, the maximum likelihood tree obtained by PHYML is also nearly identical (Additional file 1). Interestingly, the tree contains a single clade with 216 non-fungal sequences and four fungal clades containing 971, 785, 213 and 18 sequences each. This corresponds to the fact that many fungi have various paralogs. Since the fungal sequences are clearly separated from the non-fungal eukaryotic sequences it appears the evolutionary rate of fungal sedolisins has been higher than that of other sedolisins. A process of functional redundancy, caused by gene duplications, and a resulting functional diversification, corresponds with an increased evolutionary rate.

It is possible that the hypothesized disulfide bridge C402-C452 in the SED_A model has played a crucial role in the evolution of fungal sedolisins. First, we consider the absence of the bridge in the SED_A model as a modeling artifact. The fact that two cysteines are modeled within 5 Å distance combined with the fact that they are strictly conserved among fungal sedolisins, is not likely a mere coincidence. Then, it can be envisaged that such a disulfide bridge stabilizes the enzyme in the oxidative extracellular environment. A more stable enzyme is more robust and allows for accelerated evolution [49]. The predicted bridge appears to be required for the conformational change near the binding pocket as is shown by the virtual mutant (Fig. 4c). Furthermore it is striking that C402 of SED_A corresponds with position 341 in 3EE6, very close to many of the key SDPs. An obvious requirement for endosedolisin diversification would be the presence of at least part of CSI2 since the presence of the disulfide bond in SED_B does not affect the conformation, suggesting its presence is not sufficient for the conformational change in SED_A. Another possible explanation for the accelerated evolutionary rate in fungi, also related with the novel disulfide bridge, is given by the fact that fungal sedolisins are predicted to be secreted, hence act in a highly variable environment, whereas human TPP is lysosomal, which puts a rather high functional constraint.

The CSIs form a major obstacle when studying diversification of fungal sedolisins. Not only does their presence negatively affect the alignment process, it will also affect folding, making modeling less reliable. The residue quality (resQ) scores provided by I-TASSER give an indication of the reliability of the model and confirm that the CSI regions are unreliably modeled whereas in general confidence of the models is good (e.g. see for model of SED_A in Fig. 4a and b). The fact that the loops are likely incorrectly modeled does however not imply that their approximate location is incorrect and we envisage their solvent exposed location does not severely affect the folding of the core. This is supported by the dimer modeling of SED_A without CSI1 that, as part of the propeptide, is supposedly removed during zymogen activation (Additional file 2). It is difficult to envisage or explain how CSIs 1, 3 and 4 are related with the hypothesized diversification towards endosedolisin and TPP but the fact that CSI2 seems to instigate a conformational change and that removing CSI2 from SED_A yields a model that resembles the fold of 3EE6 (Fig. 4) is intriguing. Furthermore, CSI2 and CSI4 are conserved among all and most Hypo-endosedolisins respectively (Fig. 2 b d) and are located on opposite sides of the predicted pocket cavity of Hypo-endosedolisins. As such they might affect the exact conformation of the pocket. They also may have some importance in substrate binding or contain retention signals. Interestingly CSI2 is the only CSI that corresponds perfectly with the clustering into Hypo-endo and Hypo-TPP. CSI3 and CSI1 show no clear conservation and are found near to the calcium binding site and in the prosegment respectively and there are no clues regarding their functions.

A number of approaches and softwares to predict SDPs exist, although it must be noted that most often CDPs are identified. CDPs are most likely the result of positive selection but neutral evolution, or even phylogenetic reconstruction artifacts, can also result in CDP identifications. Diverge [50] should theoretically take this into account by using likelihood models. A recent version of Evolutionary Trace [51] includes mutual information in order to substantiate its predictions. We combined SDPfox [36], to identify CDPs, with MISTIC [37], to confirm CDPs as SDPs. In a recent paper we showed that that combination identified a number of known target positions in the evolution of truncated hemoglobins [52], which verifies the applicability of the method. The major disadvantage of MI is the requirement of large datasets. Then, given the high noise neutral evolution combined with epistasis can provide, robust MI determination requires many corrections or adjustments, such as provided by MISTIC. However, MISTIC’s output consists of z-scores that tend to increase with the number of sequences. We used the default cut-off threshold of 6.5, which was determined for using sets of about 400 sequence clusters. The CDP network we obtained from the Hypo-Endo / Hypo-TPP dataset contained 533 sequence clusters by which a more stringent threshold should be applied. Unfortunately, there is no easy method for the determination of that threshold. The separate Hypo-Endo and Hypo-TPP analysis had 214 and 300 sequence clusters each, by which the cut-off should likely be applied at a lower threshold. Using the 6.5 threshold, we identified SDN1 using the Hypo-TPP dataset and SDN2 and SDN3 using the Hypo-Endo dataset. Figure 6 clearly shows that not only SDN1 is similar to SDN2 sharing, what we refer to as, five key SDPs, but also that that three of these show the highest levels of connectivity to other SDPs in the same networks. Hence, we used a high threshold, identified similar networks using independent datasets yielding a number of highly connected SDPs. Basically, the method we used here is therefore specific rather than sensitive and as such we are particularly confident in the five key SDPs.

We have no explanation for SDN3 that shares the highly connected SDP228 with SDN1, SDN2 and SDN3 only connect when applying a very low threshold. All together, out of the 26 CDPs we selected only 15 SDPs and 1 supportive pSDP, besides 5 pSDPs for which no corroborating evidence was found. Hence, this further corroborates the method suffers from poor sensitivity rather than poor specificity.

The advantage in using SDNs is not only reflected in that it allows for a substantiation of SDP prediction, it also relies in the fact that it shows co-evolving partners allowing a more elaborated explanation and improved predictions and understanding of the complete process of functional diversification. As stated before, based on the tree topology we envisaged a single functional diversification towards, based on biochemical evidence, endosedolisin and TPP activity. Although there are many uncertainties since it seems many factors, including SDPs but also at least one of four CSIs, are involved, most results seem to converge around the conformational change of Helix 9 identified in the SED_A model. The endosedolisin activity seems not only caused by additional helix 9b, coded by CSI2, but is also related with the interaction with the chaperone-like prosegment, as shown by the apparently important interaction between the aliphatic sidechain of K346 and F89, further strengthened by SDP73. Models of virtual mutants directed at the central role of SDP307 residing in helix 9, the compensations of additional key SDPs 89, 343, 346 and 349, the predicted disulfide bridge and also the four CSIs (Fig. 4) suggest all are involved in that conformational change. Then, if we consider the conservation pattern of Trp/Phe of SDP307 in the hypo-TPP clade, we should actually consider that position 307 is an SDP in the TPP clade. Thus, although the evolutionary processes have taken place in two clades and are therefore strictly independent, they seem to converge at a central role for SDP307. Correspondingly, W307 has been shown to be important for activity in human TPP [15] and the same position is identified as the major difference of the clade containing 213 uncharacterized sedolisins. Since this clade contains at least both asco- and basidiomycete sequences, this clade is unlikely the result of a neutral mutation, by which it is likely the result of yet another as yet unknown functional diversification.

Although SDPs, mutant analysis and literature confirm each other, future validation of the proposed functional differences requires molecular dynamics and or wetlab experiments. Given the sheer amount of characteristics (i.e. CSIs and SDPs) involved, this will be difficult to achieve. As such, maybe a more feasible approach is to obtain a structure of SED_A or another characterized endosedolisin such as Aorsin.

The remaining question concerns how this change in conformation is related to the difference in activity. TPP activity can be envisaged to require a less spacious binding cleft, as can be seen by comparing Fig. 4d and e, in concordance with the fact that SDP307 is occupied by a large hydrophobic residue in the Hypo-TPP and NF clades and by small hydrophobic in Hypo-Endo.

## Conclusion

Fungal sedolisins have undergone a process of birth and death evolution [53] or, more exactly, a process of functional redundancy and diversification. Diversification has resulted in TPP and endopeptidase subfamilies, as was suggested by Reichard and coworkers [23]. In the endopeptidase subfamily, a CSI seems to be involved in a conformational change, likely possible by the predicted disulfide bridge identified in all fungal sedolisins. A network of SDPs, particularly the local network of SDPs 340–343–346-89 and the more distant SDP307 appear to be involved in the hypothesized diversification towards endo activity whereas other SDPs are linked to TPP activity. The interaction between SDP89 and SDP346 correspond with the chaperone-like character of the large prosegment of sedolisins. Besides the corroboration of predicted SDPs, MI network analysis confirms the generally accepted idea that evolution towards two specificities is independent and shows that MI analysis, albeit powerful, should be performed with care.

## Additional files

Additional file 1: Comparison of PHYML (Left) and Consensus Bootstrap FastTree (Right). Two sequences indicated by arrows in the FastTree show a major change in clade position. (TIF 386 kb)

Additional file 2: Modeling of SED_A dimer (A) Cartoon of dimer with all CSI’s; (B) Cartoon of dimer without CSI1, likely part of propeptide. In metallic green cartoon the core, in metallic pink cartoon the prosegment, in blue and white wireframe the CSI’s as part of the two monomers. Monomers were structurally aligned to the 3EE6 dimer (not shown). (C) Cartoon of 3EE6 dimer interface mediated by Zn ions (red spheres). Zn576 of chain A (left, style and color as in A and B) interacts with H197 and D457 from chain A and E529 from chain B (right). Distances between the residues are 3.12 Å (H197-D457); 4.25 Å (H197-E529); and 4.06 Å (D457-E529). Similarly, Zn 577 of Chain B interacts with H197 and D457 from chain B and E529 from chain A, thereby forming a dimer. The six interface residues are indicated in red wireframe. Residues 180 and 197, neighboring the removed linker peptide, are in orange sphere. (D) Circo presentation of MI analysis Hypo-Endo dataset. Red and Black connections have high (Top 5%) and medium (70–95%) MI respectively. (TIF 2778 kb)

Additional file 3: Cartoon showing location of SDPs. Red wireframe: Catalytic site; Orange wireframe: Oxyanion; Blue wireframe: Key SDPs; Green wireframe: Other SDPs; Yellow wireframe: Other CDPs including pSDPs. (TIF 685 kb)

Additional file 4: Modeling of Cysteines 402 and 452 from SED_A, strictly conserved among all fungal sedolisins and absent in non-fungal sedolisins. (TIF 523 kb)

Additional file 5: Complete Multiple Sequence Alignment. (FAA 3690 kb)

## Abbreviations

CDP: Cluster Determining Position
CSI: Cluster Specific Insert
MI: Mutual Information
MSA: Multiple Sequence Alignment
SDN: Specificity Determining Network
SDP: Specificity Determining Positions
TPP: TriPeptidyl Peptidase
WT: wild type
