# Supplementary figures and images for: Structure-function analysis of Sedolisins: evolution of tripeptidyl peptidase and endopeptidase subfamilies in fungi
Source: BMC Bioinformatics. 2018 Dec 4;19:464. doi: 10.1186/s12859-018-2404-y (PMC6278154; doi:10.1186/s12859-018-2404-y)

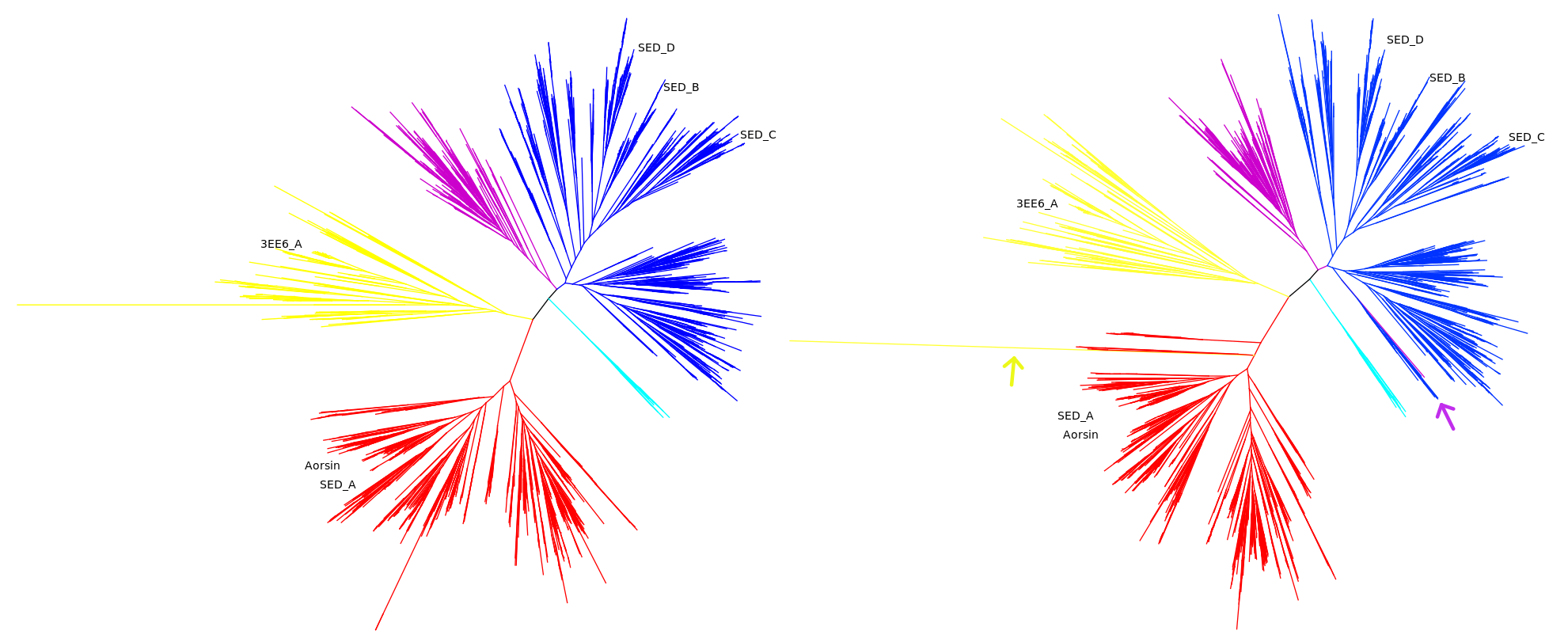

Supplement: Supplementary file 1 — Comparison of PHYML (Left) and Consensus Bootstrap FastTree (Right). Two sequences indicated by arrows in the FastTree show a major change in clade position. (TIF 386 kb) [file 12859_2018_2404_MOESM1_ESM.tif]

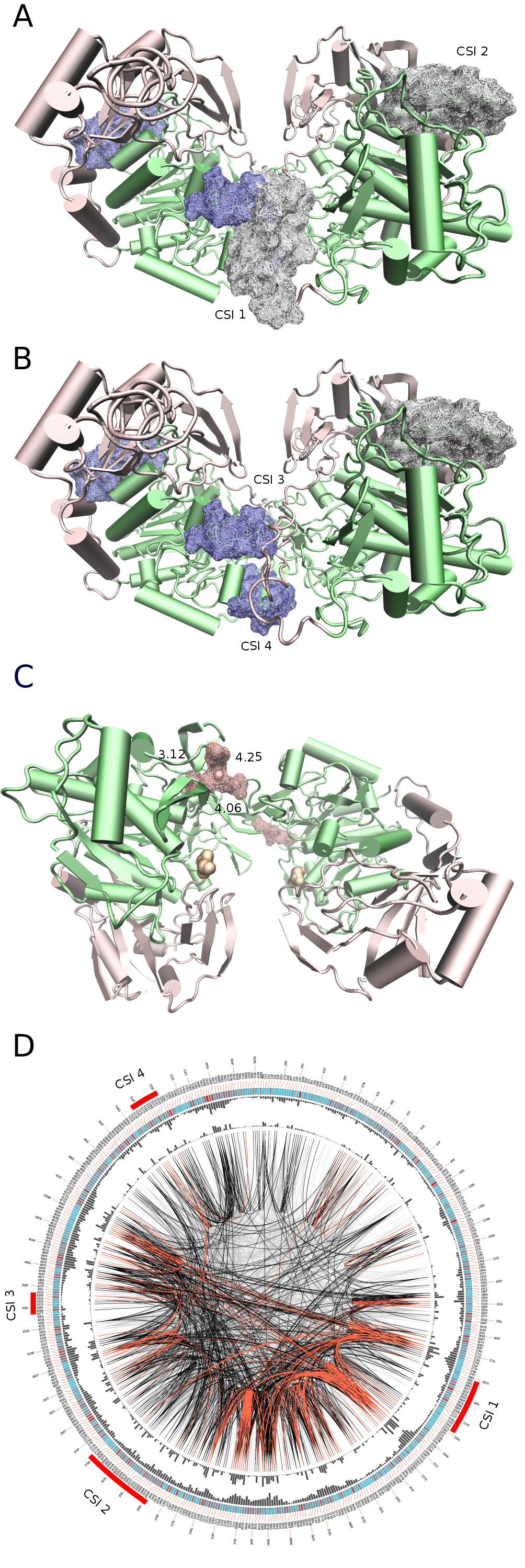

Supplement: Supplementary file 2 — Modeling of SED_A dimer (A) Cartoon of dimer with all CSI’s; (B) Cartoon of dimer without CSI1, likely part of propeptide. In metallic green cartoon the core, in metallic pink cartoon the prosegment, in blue and white wireframe the CSI’s as part of the two monomers. Monomers were structurally aligned to the 3EE6 dimer (not shown). (C) Cartoon of 3EE6 dimer interface mediated by Zn ions (red spheres). Zn576 of chain A (left, style and color as in A and B) interacts with H197 and D457 from chain A and E529 from chain B (right). Distances between the residues are 3.12 Å (H197-D457); 4.25 Å (H197-E529); and 4.06 Å (D457-E529). Similarly, Zn 577 of Chain B interacts with H197 and D457 from chain B and E529 from chain A, thereby forming a dimer. The six interface residues are indicated in red wireframe. Residues 180 and 197, neighboring the removed linker peptide, are in orange sphere. (D) Circo presentation of MI analysis Hypo-Endo dataset. Red and Black connections have high (Top 5%) and medium (70–95%) MI respectively. (TIF 2778 kb) [file 12859_2018_2404_MOESM2_ESM.tif]

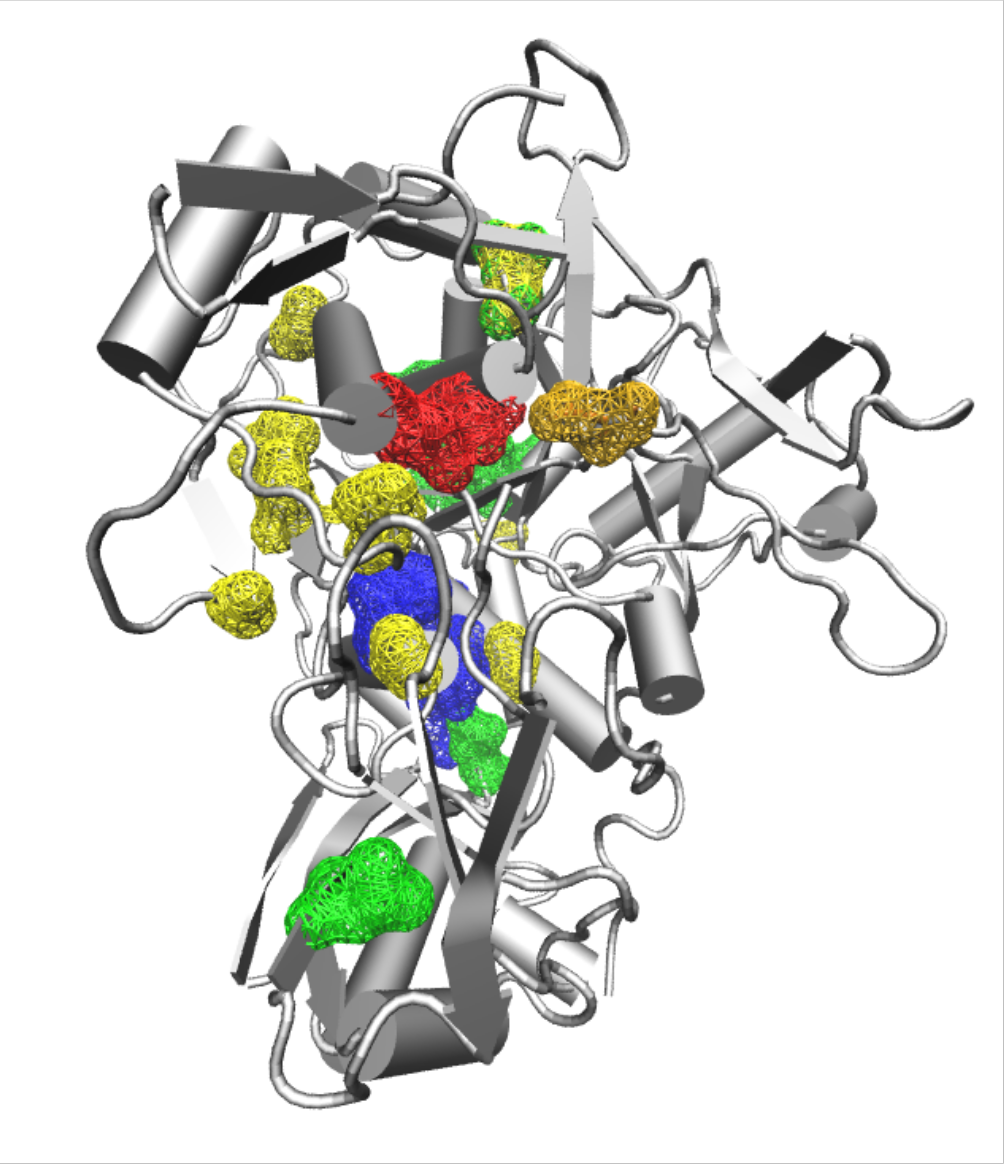

Supplement: Supplementary file 3 — Cartoon showing location of SDPs. Red wireframe: Catalytic site; Orange wireframe: Oxyanion; Blue wireframe: Key SDPs; Green wireframe: Other SDPs; Yellow wireframe: Other CDPs including pSDPs. (TIF 685 kb) [file 12859_2018_2404_MOESM3_ESM.tif]

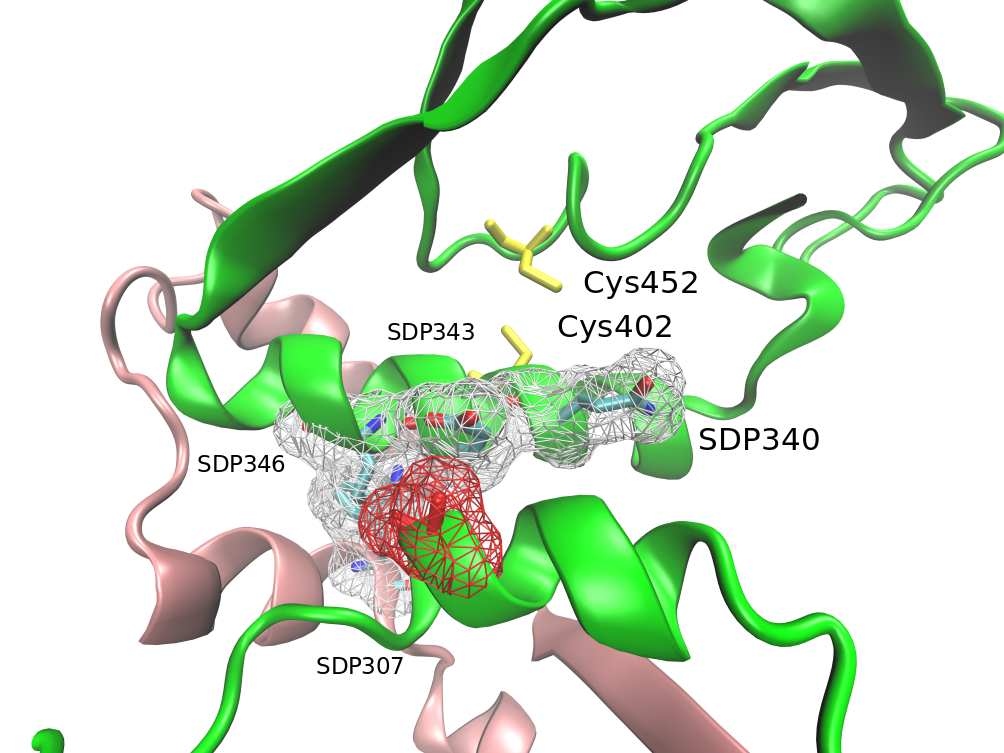

Supplement: Supplementary file 4 — Modeling of Cysteines 402 and 452 from SED_A, strictly conserved among all fungal sedolisins and absent in non-fungal sedolisins. (TIF 523 kb) [file 12859_2018_2404_MOESM4_ESM.tif]
